# Determining Adult Dizziness and Sleep Quality After the 2023 Earthquakes in Türkiye

**DOI:** 10.7759/cureus.64632

**Published:** 2024-07-16

**Authors:** Tulin Gesoglu Demir, Suzan Havlioğlu

**Affiliations:** 1 Neurology, Harran University Faculty of Medicine, Sanliurfa, TUR; 2 Public Health/Nursing, Harran University Faculty of Health Sciences, Sanliurfa, TUR

**Keywords:** vertigo, sleep disorders, dizziness, disaster, turkey - syria earthquake

## Abstract

Background: This study investigated the presence of dizziness and sleep quality in people affected by the earthquake disaster of 2023 in Türkiye and aimed to determine the relationship between dizziness and sleep disorders.

Methods: A total of 384 earthquake survivors who had no previous complaints of dizziness or sleep disturbance and who presented to the neurology outpatient clinic with complaints of dizziness or sleep disturbance after the earthquake were included in the study. Demographic information of participants and scales such as the Dizziness Handicap Inventory (DHI) and the Pittsburgh Sleep Quality Index (PSQI) were recorded.

Results: It was determined that the majority of the patients in the 18-35 age group had poor sleep quality and there was a significant difference in the PSQI score. Additionally, a moderate positive significant relationship was found between the DHI score and the PSQI score.

Conclusion: Exposure to major earthquakes and aftershocks can cause sleep disorders and dizziness complaints, which may emerge as public health problems. Earthquake victims should be followed up and necessary interventions should be made early.

## Introduction

In the early morning of February 6, 2023, a 7.8 magnitude earthquake struck southeastern Türkiye and some parts of Syria. Approximately nine hours later, it was followed by another 7.5 magnitude earthquake and numerous aftershocks [1]. The two consecutive earthquakes affected 9.1 million people in 11 provinces, caused 44,000 deaths and 108,000 injuries, and displaced 2.2 million people [2]. The period following any strong earthquake has been associated with various types of organic and non-organic diseases and disorders. Myocardial infarction and stroke [3], sleep disorders [4], increased suicidal ideation [5], and dizziness [6-7] are increasingly common among survivors of major earthquakes. A cross-sectional study conducted in the cities of Dujiangyan, Beichuan, and Qingchuan in China’s Sichuan province revealed an 83.2% prevalence of sleep disorders among earthquake survivors [8]. A cohort study additionally showed that nearly half of adolescent survivors in Dujiangyan continued to have sleep disorders 2.5 years after the earthquake [9].

Severe earthquakes also occasionally cause varying degrees of dizziness [6,10]. Dizziness after an earthquake is thought to relate to the potential effects of autonomic stress on balance function and/or psychological factors [6]. After the earthquake in Nepal in April and May 2015, the number of patients with vague vestibular features (e.g., imbalance and dizziness) not in any category of vestibular disorders increased [10]. Another report found that poor sleep quality was associated with exacerbation of vertigo symptoms in patients with psychogenic dizziness, such as phobic postural vertigo and chronic subjective dizziness or nonspecific dizziness [11].

The consequences of natural disasters can strike immediately and/or develop in the years that follow [9]. Dizziness and sleep disorders that may occur in addition to an earthquake’s other devastating effects may persist for years and can be regarded as an earthquake-related public health problem. Although several studies have separately examined post-earthquake dizziness [6,7,12] and sleep disorders [4,13] separately, none have evaluated the relationship between dizziness and sleep disorders among earthquake victims. In response, we investigated the presence of dizziness and level of sleep quality among people affected by the February 6 earthquakes in Turkey in order to determine the relationship between the two conditions.

## Materials and methods

This was a cross-sectional, descriptive study conducted at the Department of Neurology, Harran University Faculty of Medicine, Şanlıurfa, Türkiye, to determine the presence of dizziness and level of sleep quality among adults who have experienced an earthquake. Participants were recruited from the outpatient clinic of the study hospital between July and August 2023. Recruitment was conducted by inviting individuals who visited the clinic during this period and met the study criteria. The study was approved by the Ethics Committee of Harran University Faculty of Medicine (approval number: HRU/23.11.16 dated June 19, 2023).

Inclusion criteria

The inclusion criteria for participants were as follows: (i) Aged 18 years or older, (ii) Residents of Sanliurfa at the time of the February 6 earthquakes, (iii) Experienced the February 6 earthquakes, and (iv) No prior diagnosis of vertigo or sleep disorders.

Sampling method

The sample size was calculated to be 384 participants, based on the population size and using a standard sample size calculation formula for cross-sectional studies. The sampling targeted individuals who met the inclusion criteria and were willing to participate in the study.

Data collection

To collect data regarding sleep quality, we used a sociodemographic information form that we developed with reference to the literature, along with the Dizziness Handicap Inventory (DHI) and the Pittsburgh Sleep Quality Index (PSQI). After the participants were informed about the study, their verbal consent to participate was obtained. Data were collected in face-to-face interviews, each lasting approximately 10 minutes.

Sociodemographic Information Form

The sociodemographic information form that we developed consisted of 19 questions addressing age, sex, income status, occupation, marital status, level of education, history of psychiatric illness, place of residence at the time of the earthquake(s), financial loss or death in the family due to the earthquake(s), and experiences of dizziness or sleep disorders following the quake(s).

DHI

The DHI, developed in 1990 [14], consists of 25 items aimed at determining factors that aggravate dizziness and balance disorder, as well as physical, emotional, and functional outcomes, in cases of vestibular disorders. Questions 1, 4, 8, 11, 13, 17, and 25 examine physical disability, questions 2, 9, 10, 15, 18, and 20-23 examine emotional disability, and questions 3, 5-7, 12, 14, 16, 19, and 24 examine functional disability. Each question’s response options are yes (4 points), no (0 points), and sometimes (2 points). For scoring the items of the DHI, 28 points is suggested as the cutoff for physical disability and 36 points as the cutoff for functional and emotional disability. High scores are interpreted to indicate that the respondent’s dizziness is hindering their life at an advanced level. The Turkish version of the scale was validated and permission was obtained for the study [15].

PSQI

The PSQI was developed in 1989 [16] and adapted to the Turkish population in 1996 [17]. The PSQI is a 19-item self-report scale used to evaluate sleep quality and disturbance in the past month. The PSQI consists of 24 questions: 19 self-report questions and five questions to be answered by the respondent’s spouse or roommate. The PSQI’s 18 scored questions are grouped into seven categories: subjective sleep quality, sleep latency, sleep duration, habitual sleep efficiency, sleep disorder, sleeping drug use, and daytime dysfunction. Each category is evaluated on a scale of 0-3 points, and the total scores in each category yield the total scale score, which ranges from 0 to 21 points. A total scale score greater than 5 points indicates “poor sleep quality.” Permission to use the scale for this study was obtained from authors who adapted the scale to the Turkish population.

Statistical analysis

We statistically analyzed the data using the IBM SPSS Statistics for Windows, Version 22.0 (Released 2013; IBM Corp., Armonk, New York, United States) and evaluated whether the data conformed to a normal distribution using the Shapiro-Wilk test. To evaluate the data with normal distribution, we employed descriptive statistics (i.e., number, percentage, and mean), an independent sample t-test, an ANOVA, the Mann-Whitney U test, the chi-square test, and the Pearson correlation coefficient. All p-values less than .05 were considered to be statistically significant.

## Results

We evaluated a sample of 384 earthquake victims, 191 (49.7%) of whom were female; the mean age was 33.63±12.71 years. The participants’ sociodemographic and earthquake experience-related characteristics are listed in Table 1.

**Table 1:** Sociodemographic characteristics of the study participants (N=384)

Variables		Frequency	Percentage
Sex	Female	191	49.7
	Male	193	50.3
Age (years)	18-35	257	66.9
	36-86	127	33.1
Marital status	Married	210	54.7
	Single	161	41.9
	Other	13	3.4
Educational status	Never went to school	46	12.0
	Primary education	86	22.4
	High school	93	24.2
	University	159	41.4
Income status	Income less than expenses	185	48.2
	Income equals expenses	144	37.5
	More income than expenses	55	14.3
History of psychiatric illness	Yes	42	10.9
	No	342	89.1
Place of residence at the time of the earthquake	Rural	81	21.1
	Urban	303	78.9
Floor of the house	0-1	141	36.7
	>2	243	63.3
Was your house damaged during the earthquake?	No damage	108	28.1
	Moderate damage	235	61.2
	Severe damage	41	10.7
Have you been under a rubble?	Yes	15	3.9
	No	369	96.1
Did you stay in a tent after the earthquake?	Yes	82	21.4
	No	302	78.6
Did you change your home after the earthquake?	Yes	63	16.4
	No	320	83.3
Do you have a complaint of dizziness after an earthquake?	Yes	148	38.5
	No	236	61.5
Do you have any complaints about sleep problems after the earthquake?	Difficulty falling asleep	142	37.0
	Difficulty staying asleep	85	22.1
	Woke prematurely	75	19.5
	Difficulty waking up	51	13.3
Did your sleep duration change after the earthquake?	Yes	159	41.4
	No	225	58.6
Pittsburgh Sleep Quality Index score	Good (0-5 point)	119	31.0
	Bad (>6 point)	265	69.0

After the earthquakes, 159 (41.4%) participants stated that their sleep duration changed, 142 (37.0%) had difficulty falling asleep, 85 (22.1%) had difficulty staying asleep, 75 (19.5%) woke prematurely, and 51 (13.3%) had difficulty waking up. The mean sleep duration of the participants was 6.52±1.54 hours. A total of 148 (38.5%) participants complained of dizziness after the earthquake and 265 (69.0%) had poor sleep quality after the earthquake. The participants’ average scores in the DHI's subunits and the PSQI’s categories appear in Table 2.

**Table 2:** Participants' PSQI and DHI mean scores PSQI: Pittsburgh Sleep Quality Index, DHI: Dizziness Handicap Inventory

Scale Total and Sub-Dimensions	Minimum	Maximum	Mean	Standard Deviation
Physical dimension of DHI	0	28	8.80	8.45
Sensory dimension of DHI	0	36	7.42	9.37
Functional dimension of DHI	0	36	10.33	10.93
DHI Total	0	100	26.61	27.39
Subjective sleep quality	0	3	1.29	0.77
Sleep latency	0	3	1.49	0.85
Sleep time	0	3	1.29	0.98
Habitual sleep activity	0	3	0.53	0.86
Sleeping disorder	0	3	1.39	0.74
Sleeping medicine use	0	3	0.26	0.66
Daytime sleep dysfunction	0	3	1.20	0.99
PSQI Total	0	18	7.48	3.39

**Figure 1:**
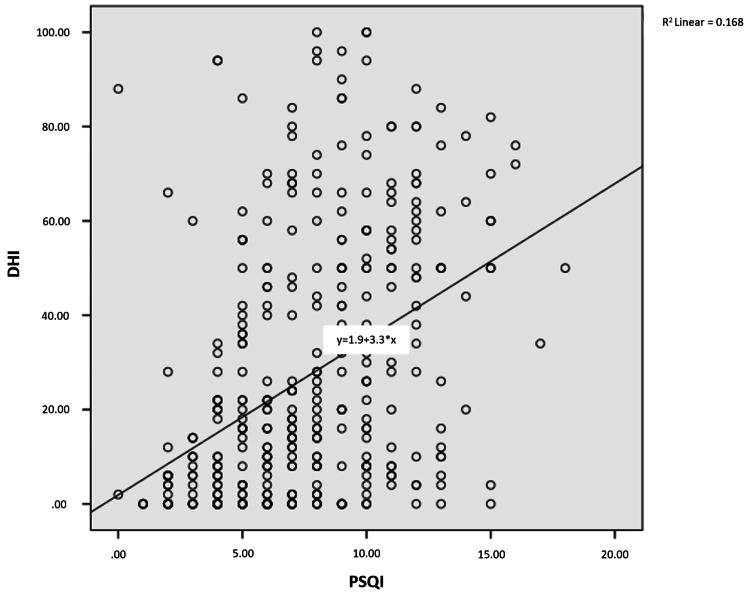
Relationship between PSQI and DHI scores PSQI: Pittsburgh Sleep Quality Index, DHI: Dizziness Handicap Inventory

**Figure 2:**
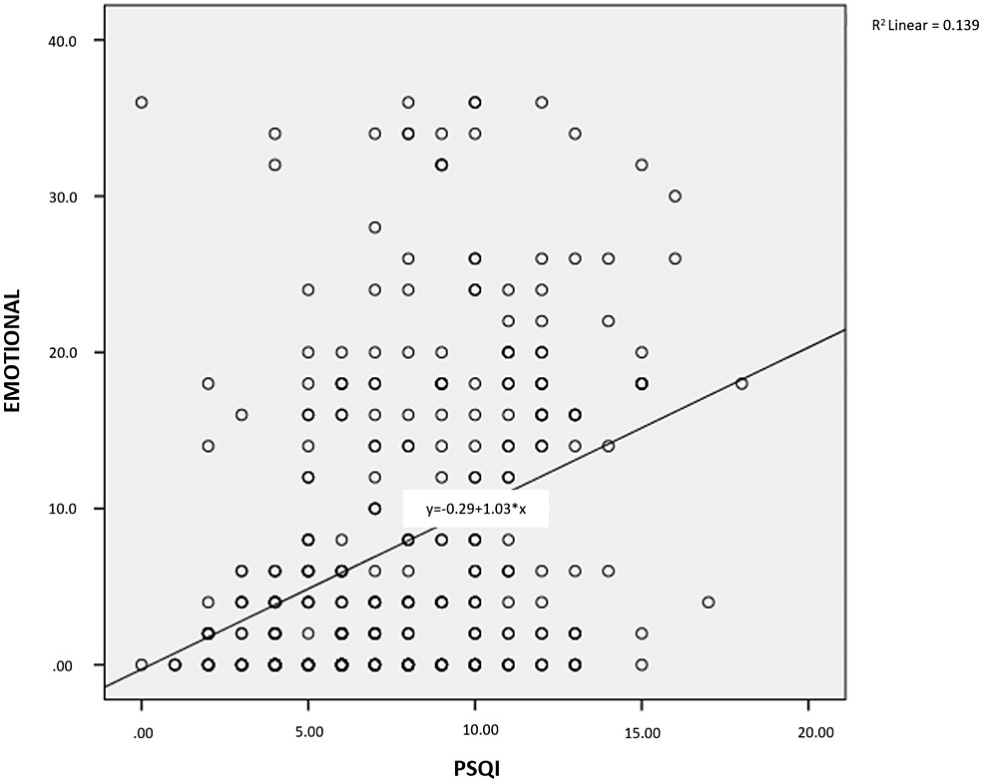
Relationship between PSQI score and emotional disability PSQI: Pittsburgh Sleep Quality Index

**Figure 3:**
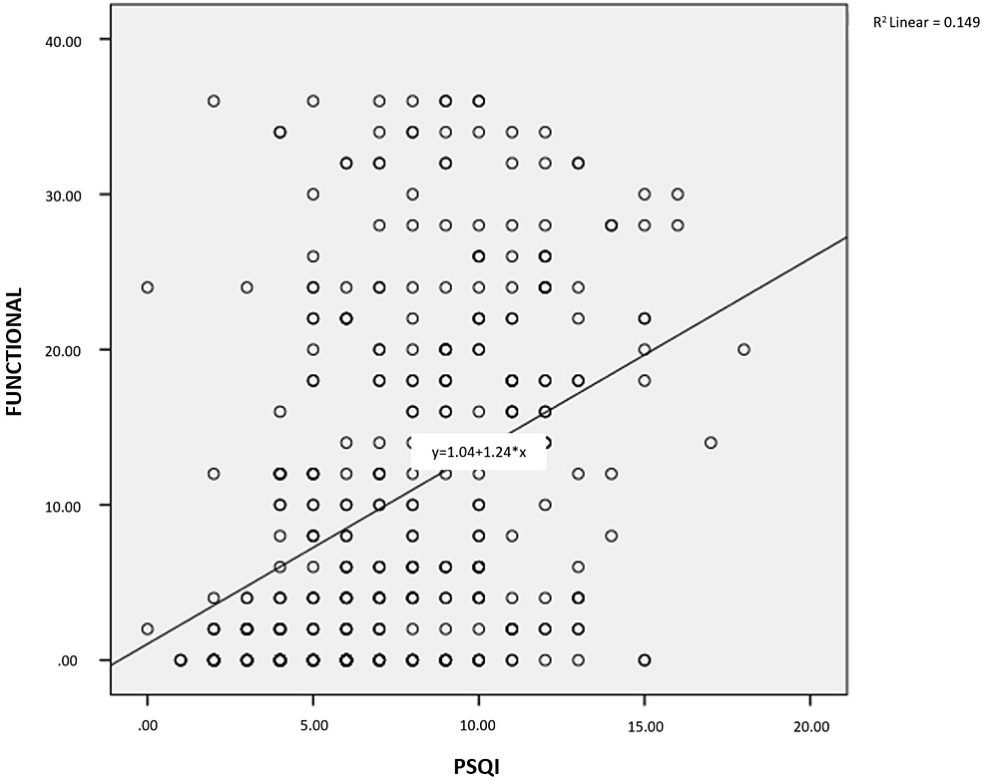
Relationship between PSQI score and functional disability PSQI: Pittsburgh Sleep Quality Index

**Figure 4:**
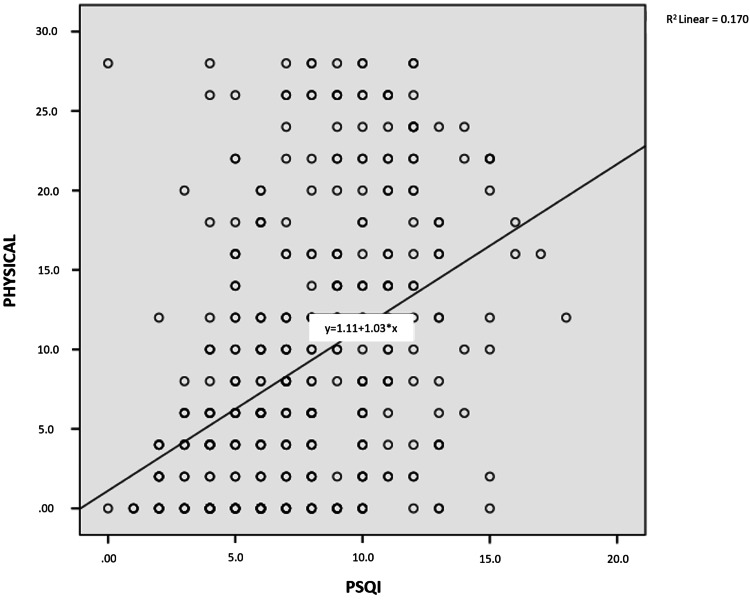
Relationship between PSQI score and physical disability PSQI: Pittsburgh Sleep Quality Index

As shown in Figures 1-4, a weak, positive significant relationship emerged between participants’ physical disability, emotional disability, and functional disability and the PSQI score, on the one hand, and, on the other, their DHI and PSQI scores (p<.05).

Table 3 allows a comparison of the participants’ sociodemographic data and PSQI scores. Overall, 187 (72.8%) of the 18-35-year-old age group had poor sleep quality, and that age group had PSQI scores that differed significantly from scores in the other age groups (p=.024).

**Table 3:** Comparison of participants' sociodemographic characteristics and PSQI scores * Pearson chi-sguare test PSQI: Pittsburgh Sleep Quality Index

Variable		PSQI points				p-value
		Good Sleep Quality (PSQI < 5)		Poor Sleep Quality (PSQI > 5)		
		Frequency	Percentage	Frequency	Percentage	
Sex	Female	56	29.3	135	70.7	.481*
	Male	63	32.6	130	67.4	
Age (years)	18-35	70	27.2	187	72.8	.024*
	36-86	49	38.6	78	61.4	
Marital status	Married	72	34.3	138	65.7	.125*
	Single and other	47	27.0	127	73.0	
Educational status	Never went to school	20	43.5	26	56.5	.129*
	Primary education	29	33.7	57	66.3	
	High school	29	31.2	64	68.8	
	University	41	25.8	118	74.2	
Income status	Income less than expenses	50	27.0	135	73.0	.252*
	Income equals expenses	51	35.4	93	64.6	
	More income than expenses	18	32.7	37	67.3	
History of psychiatric illness	Yes	10	23.8	32	76.2	.286*
	No	109	31.0	233	69.0	
Place of residence at the time of the earthquake	Rural	31	38.3	50	61.7	.111*
	Urban	88	29.0	215	71.0	
Floor of the house	0-1	53	37.6	88	62.4	.033*
	>2	66	27.2	177	72.8	
Was your house damaged during the earthquake?	No damage	44	40.7	64	59.3	.003*
	Moderate damage	70	29.8	165	70.2	
	Severe damage	5	12.2	36	87.8	
Have you been under rubble?	Yes	5	33.3	10	66.7	.841*
	No	114	30.9	255	69.1	
Did you stay in a tent after the earthquake?	Yes	20	24.4	62	75.6	.145*
	No	99	32.8	203	67.2	
Did you change your home after the earthquake?	Yes	12	19.0	51	81.0	.025*
	No	107	33.3	214	66.7	
Do you have a complaint of dizziness after an earthquake?	Yes	35	23.6	113	76.4	.014*
	No	84	35.6	152	64.4	
Did your sleep duration change after the earthquake?	Yes	28	17.6	131	82.4	.001*
	No	91	40.4	134	59.6	
Do you have any complaints about sleep problems after the earthquake?	Yes	85	27.2	228	72.8	.001*
	No	34	47.9	37	52.1	

Participants whose houses had two or more floors during the earthquakes (n=177, 72.8%) and whose houses were moderately or severely damaged (n=36, 87.8%) had poor sleep quality and significantly different PSQI scores (p = .033 and p = .033, respectively). Poor sleep quality affected 113 (76.4%) participants who complained of dizziness after the earthquakes, 131 (82.4%) participants whose sleep duration changed, and 228 (72.8%) participants who had sleep disorders. Significant differences also emerged between PSQI score, on the one hand, and, on the other, complaints of dizziness (p = .014), changes in sleep duration (p = .001), and sleep problems (p = .001), as shown in Table 3. While these differences reached statistical significance, it is important to note that some of the absolute percentage differences among the comparison subgroups were relatively small.

Table 4 allows a comparison of the participants’ sociodemographic data and DHI scores. Women had higher DHI scores than men (p<.05), and participants whose houses were moderately or severely damaged by the earthquakes had significantly higher scores than other participants (p < .05). Participants whose sleep duration changed and who had complaints about sleep disorders after the earthquakes also had significantly higher DHI scores than other participants (p < .05). No statistically significant difference surfaced between age, marital status, income level, and floor of residence and DHI scores (p > .05).

**Table 4:** Comparison of participants' sociodemographic characteristics and DHI scores *Independent simple t test, **Anova, ***Mann-Whitney U test DHI: Dizziness Disability Handicap

Variable		Mean	Standard deviation	p-value
Sex	Female	29.62	29.61	.032*
	Male	23.62	24.69	
Age (years)	18-35	25.66	26.26	.335*
	36-86	28.53	29.54	
Marital status	Married	26.43	26.83	.889*
	Single and other	26.83	28.13	
Educational status	Never went to school	39.60	34.00	.001**
	Primary education	30.58	28.92	
	High school	25.54	25.72	
	University	21.30	23.80	
Income status	Income less than expenses	27.34	28.06	.505**
	Income equals expenses	27.21	26.62	
	More income than expenses	27.23	27.23	
Place of residence at the time of the earthquake	Rural	35.75	29.78	.001*
	Urban	24.16	26.22	
Floor of the house	0-1	29.74	28.72	.097*
	>2	24.81	26.48	
Was your house damaged during the earthquake?	No damage	22.05	25.07	.001**
	Moderate damage	26.20	26.92	
	Severe damage	40.87	31.52	
Did you stay in a tent after the earthquake?	Yes	34.97	31.79	.002*
	No	24.33	25.65	
Did you change your home after the earthquake?	Yes	38.92	31.87	.001*
	No	24.19	25.79	
Do you have a complaint of dizziness after an earthquake?	Yes	35.35	29.33	.001*
	No	21.11	24.61	
Did your sleep duration change after the earthquake?	Yes	32.74	27.80	.001*
	No	22.31	26.31	
Do you have any complaints about sleep problems after the earthquake?	Yes	28.83	27.28	.001*
	No	16.84	25.84	
		Mean Rank		
History of psychiatric illness	Yes	254.20		0.001***
	No	184.34		
Have you been under rubble?	Yes	305.93		0.001***
	No	187.36		

## Discussion

Large earthquakes are commonly associated with sleep disorders and dizziness [4,6,7]. In our study, we investigated the frequency of dizziness and sleep disorders among individuals exposed to two consecutive major earthquakes and the relationship between the two conditions. In our results, the prevalence of dizziness and sleep disturbance after the earthquakes was high, and there was a weak correlation between dizziness and sleep disorders. 

After the earthquakes, there were changes in the sleep duration of the participants; 37% had difficulty falling asleep, 22.1% had difficulty staying asleep, 19.5% woke up early, and 13.3% had difficulty waking up. In a different sample of 1,573 earthquake survivors, approximately 50% of participants reported sleeping less than seven hours per night, 28% had difficulty falling asleep, 40% experienced daytime fatigue, and 23% reported poor sleep quality [18]. In another similar study, 18.8% of participants had poor sleep quality, 7.4% had difficulty falling asleep, and 7.1% had difficulty staying asleep [19].

In our study, 187 (72.8%) of the 18-35-year-old age group had poor sleep quality and PSQI scores that differed significantly from scores in the other age groups (p = .024). In a different study evaluating 999 earthquake victims, significant differences in sleep quality surfaced between different age groups, while average sleep quality decreased as PSQI scores and age increased [20]. In another study involving 6,132 earthquake-affected individuals aged 9-18 years old, older participants had a significantly higher risk of sleep disorders than younger ones [21]. Many of those problems, including poor sleep quality, difficulty falling asleep, sleeping less than seven hours, and daytime dysfunction have been reported to increase with age.

In our study, 265 (69.0%) participants had poor sleep quality. After the 2008 earthquake in Wenchuan, China, 83.2% of the survivors who were still in temporary shelter camps approximately two years later reported sleep problems, and 79.3% of them reported insomnia as their chief symptom [22]. A cohort study conducted on 1,573 adolescent survivors of that same earthquake revealed a 22.6% prevalence of poor sleep quality [18]. In another study including 999 earthquake survivors living in temporary tents and camps 10 days after a major earthquake, 20.61% of survivors had poor sleep quality while sleep latency had a positive, significant relationship with stress, and sleep disturbance had a positive, significant relationship with depression and stress [20]. The differences between our results and past findings may be due to differences in the time elapsed between the earthquake(s) and data collection.

Large earthquakes cause varying degrees of dizziness. A possible explanation for dizziness following earthquakes is that psychological stress causes balance disorders. However, another plausible hypothesis concerning why imbalance results from repeated exposure to aftershocks is that frequent physical shaking can directly disrupt the functioning of the semicircular canal system [6]. A study conducted in 2014 found that the prevalence of post-earthquake dizziness was not associated with a history of vertigo [23]. In support, our study, which included patients without vertigo before the earthquakes, revealed that 148 (38.5%) earthquake victims started to complain of dizziness after the quakes. Concerning DHI scores, female participants had a significantly higher mean score (p = .032) than males, but no significant difference emerged between the various age groups. Along similar lines, another study on complaints of dizziness subsequent to an earthquake revealed that being female was significantly associated with the onset of dizziness [24].

Poor sleep quality is associated with exacerbation of dizziness symptoms in cases of chronic dizziness involving psychological factors [11]. In our study, it was determined that those who had complaints about sleep problems after the earthquake scored significantly higher on the DHI (p < .05). PSQI and DHI scores of the patients who had sleep problems after the earthquake and whose sleep duration changed were significantly higher than the other groups (p < .05).

In a cross-sectional study of healthy individuals who experienced the 2016 earthquakes and aftershocks in Kumamoto, Japan, 1,543 participants experienced post-earthquake dizziness. The floor the individual was on at the time of the earthquakes was significantly associated with the onset of post-earthquake vertigo [24]. In our study, 177 (72.8%) of the participants living on the second or higher floor during the earthquakes had poor sleep quality. A notable discrepancy was observed between the participants' residential locations and their PSQI scores (p = .033), whereas no significant differences were identified in DHI scores (p > .05).

The post-earthquake period has been associated with the exacerbation of psychological disturbances among survivors of major earthquakes. Sleep disorders [4], anxiety disorders [25], mood disorders [26], and post-traumatic stress disorder [25] increase in prevalence after earthquakes. Although we did not evaluate post-earthquake psychological disorders in our study, the DHI scores of participants with any pre-earthquake history of psychiatric illness were significantly higher than the scores of other participants (p < .05). Even so, no significant difference between such history and PSQI scores was observed (p > .05).

One of the few studies investigating sleep quality among patients with dizziness has underscored a relationship between sleep quality and some disease subtypes associated with dizziness (i.e., benign paroxysmal positional vertigo, Ménière disease, vestibular neuritis, vestibular migraine, and psychogenic dizziness) [11]. No similar study has been conducted among earthquake victims, however. In our study, the difference between complaints of dizziness after the earthquake and PSQI scores was significant (p = .014). At the same time, a moderate, positive, significant relationship appeared between physical disability, emotional disability, functional disability, DHI score, and PSQI score. In another study, a significant correlation was observed between the severity of sleep disorders and dizziness in patients with dizziness and sleep disorders together [27]. Another study has revealed that the presence of sleep disorders in patients with chronic dizziness may relate to the decreased quality of life due to dizziness, not the actual symptom of dizziness [28].

Limitations

Our study’s findings have some limitations. Firstly, because all measurements were based on self-report, our results should be interpreted with caution. Secondly, because the study was cross-sectional, pre-earthquake and post-earthquake data could not be compared. Thirdly, we did not investigate the sequence of the onset of dizziness and sleep disturbance. Lastly, our sample consisted of patients who agreed to answer the scale questions (we, however, took only verbal informed consent and not written). Patients who refused to answer the questions were excluded from the study and, hence, we may have missed out on data from the patients who did not participate. By taking those limitations into consideration, future studies on the health effects of earthquakes can be more comprehensive and, in turn, useful for practitioners and policymakers.

## Conclusions

In our study, 265 (69.0%) earthquake victims had poor sleep quality while 148 (38.5%) began having complaints of dizziness after the earthquakes. Moreover, a weak, positive, significant relationship emerged between DHI and PSQI scores. Our results suggest that sleep disorders and dizziness can arise following exposure to major earthquakes and aftershocks and may emerge as public health problems subsequent to earthquakes. For that reason, along with responses to the immediately devastating effects of earthquakes, earthquake victims should definitely be referred to psychiatry follow-up and monitored, and necessary interventions should be implemented as early as possible.
